# Natural Macromolecules as Carriers for Essential Oils: From Extraction to Biomedical Application

**DOI:** 10.3389/fbioe.2020.00563

**Published:** 2020-06-25

**Authors:** Zora Dajic Stevanovic, Elwira Sieniawska, Kazimierz Glowniak, Natasa Obradovic, Ivana Pajic-Lijakovic

**Affiliations:** ^1^Faculty of Agriculture, University of Belgrade, Belgrade, Serbia; ^2^Department of Pharmacognosy, Medical University of Lublin, Lublin, Poland; ^3^Department of Cosmetology, University of Information, Technology and Management in Rzeszow, Rzeszow, Poland; ^4^Department of Chemical Engineering, Faculty of Technology and Metallurgy, University of Belgrade, Belgrade, Serbia

**Keywords:** bioavailability, matrix material, isoprenoids, capsulation, gastric digestion, reuse

## Abstract

Essential oils (EOs) and their main constituents, the terpenes, are widely studied, mostly relating to their antioxidant ability and bioactivity, such as antimicrobial, anticancer, anti-inflammatory, and range of other actions in the living systems. However, there is limited information on their bioavailability, especially upon clinical studies. Having in mind both strong biological effects and health benefits of EOs and their specific physicochemical properties (volatility, lipophilic character, low water solubility or insolubility, viscosity, expressed odor, concentration-dependent toxicity, etc.), there is a need for their encapsulation for target delivery. Encapsulation of EOs and their constituents is the prerequisite for enhancing their oxidative stability, thermostability, photostability, shelf life, and biological activity. We considered various carrier types such a (1) monophase and polyphase polysaccharide hydrogel carriers, (2) polysaccharide–protein carriers, and (3) lipid carriers in the context of physicochemical and engineering factors. Physicochemical factors are encapsulation efficiency, chemical stability under gastric conditions, mechanical stability, and thermal stability of carrier matrices. Choice of carrier material also determines the encapsulation technique. Consequently, the engineering factors are related to the advantage and disadvantage of various encapsulation techniques frequently used in the literature. In addition, it was intended to address the interactions between (1) main carrier components, such as polysaccharides, proteins, and lipids themselves (in order to form chemically and mechanically stable structure); (2) main carrier components with pepsin under gastric conditions (in order to form resistant material under gastric conditions); and (3) main carrier components with EOs (in order to enhance encapsulation efficiency), as a necessary precondition for whole process optimization. Finally, different sources for obtaining natural carrier macromolecules are surveyed, especially the agro-waste materials and agricultural and food by-products. This review article highlights the bioavailability aspects of encapsulated EOs and physicochemical and engineering factors concerning natural macromolecule carriers for their target delivery and application.

## Introduction

It is assumed that ~80% of the world's population relies on plant-based products in official and traditional medicine, where plant products make approximately one-quarter of the total pharmaceutical arsenal (Bhattaram et al., 2002). In addition, plant bioactive compounds are widely applied in pharmaceutics industry, cosmetics, food industry, and recently as fine (agro) chemicals and nutraceuticals (Bourgaud et al., 2001).

The isoprenoids are known as the largest family of plant bioactive compounds, participating in the composition of plant essential oils (EOs) in various combinations. Encapsulation of EO is a prerequisite for applying EOs because of some of their properties, such as volatility, intense odor and taste, dose-dependent toxicity, and very high biological activity. The encapsulation also prevents the EOs from degradation and transformation under digestion and protects the body from undesirable effects of EOs (Maderuelo et al., 2019). Moreover, entrapping of EOs enhances their bioavailability. Development of various techniques of encapsulation ensures wide use of EOs in pharmaceutical industry (e.g., Asbahani et al., 2015; Pandit et al., 2016; Arpagaus et al., 2018) and cosmetics (e.g., Martins et al., 2014; Carvalho et al., 2016). Engineering optimization of delivery process as a whole includes several interconnected steps: (1) choice of carrier matrix with best performances in accordance with particular delivery conditions, (2) ensuring the maximum of encapsulation efficiency, and (3) consideration of the reuse ability for carrier matrix components, such as natural polysaccharides and proteins. Polysaccharides, such as pectin, inulin, starch, cellulose, and hemicelluloses, are widely used in the form of single hydrogels as well as their blends. These macromolecules can be extracted from wastes of vegetable industrial processing by applying various chemical or enzymatic techniques (Poli et al., 2011). Polysaccharide hydrogels have been mixed with natural proteins, such as soy proteins, whey proteins, lecithin, and some others, in order to improve their chemical stability under gastric conditions for various biomedical and biotechnological applications, including EO encapsulation (Volić et al., 2018; Obradovic et al., 2019).

**Figure 1:**
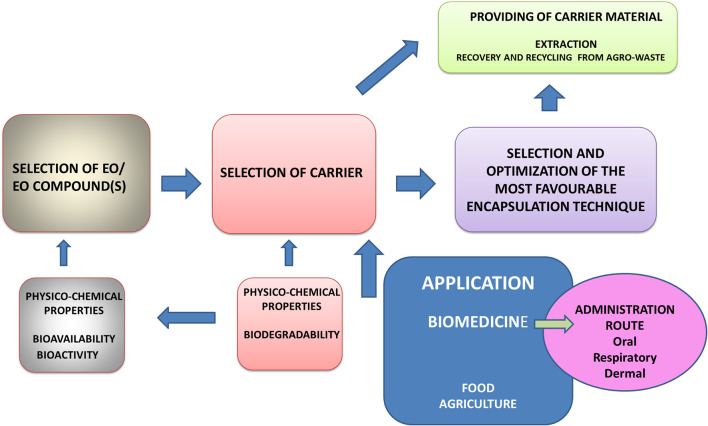
Successive steps and their interrelations in selection and optimization of carrier system for EO encapsulation.

The present article aims to review the most important groups of natural macromolecules used in entrapping of EOs in relation to the general physicochemical properties of EOs and their constituents. Our intention was also to elaborate the aspects of bioavailability and biological behavior under different administration modes, which significantly depends on the choice of carrier matrix. Performances of various carrier matrices are discussed in the context of (1) preparation procedures and (2) their mechanical and chemical stabilities under *in vivo* process conditions. The preparation procedure accounts for using various natural polysaccharides and proteins in the form of composite hydrogel matrices previously extracted from agri-food by-products. On that way, whole cycle from waste materials to various biomedical applications is elaborated on some examples in order to point out the complexity of this task. The starting point in such a complex procedure of optimization of the carrier system and encapsulation process is the characterization of bioactive core material, primarily the physicochemical features and biological behavior, as well as the application of the encapsulated product (Figure 1).

## Essential Oils—Definition, Composition, and Physicochemical Properties

Definition of International Organization for Standardization (ISO) indicates that EOs are products obtained from vegetable raw material by physical processes of distillation or pressing. The plant volatiles are typical products of aromatic plants that have been already recorded in 1,618 plant species, subspecies, or varieties representing 92 plant families according to the EssOilDB database (Kumari et al., 2014). Essential oils are complex mixtures containing mostly volatile organic compounds (VOCs) synthesized and emitted by plants in order to facilitate their growth and survival (Loreto et al., 2014). Besides VOC, EOs contain also different degradation products formed in enzymatic, chemical, or physical processes. The typical example is chamazulene formed by the breakdown of matricine during the steam distillation (Clarke, 2008). The other example is khusimone, nor-patchoulenol, or nor-tetrapatchoulol containing only 14 carbon atoms, and all formed from sesquiterpenoids (Baser and Buchbauer, 2010). Essential oils are mixtures of low-molecular-weight compounds (usually below 300 Da) and can be composed from more than a dozen, even to 300 molecules, which usually belong to 5 to 10 distinct chemical classes or congeneric groups. The content of single constituent or a congeneric group of constituents in the EOs may vary from hundredths of a percent to several dozen percent (Baser and Buchbauer, 2010; Dhifi et al., 2017).

Constituents of EOs derive from three plant biosynthetic pathways yielding isoprenoids, phenylpropanoids, and polyketides and lipids (Dudareva et al., 2006; Baser and Buchbauer, 2010; Moghaddam and Mehdizadeh, 2017). Glucose formed from carbon dioxide and water is transformed into phosphoenolpyruvate involved in the formation of phenylpropanoids (shikimates) via l-phenylalanine. The same structure of phosphoenolpyruvate after decarboxylation gives acetate, which esterifies with coenzyme-A and gives the acetyl-CoA. Polyketides and lipids are the result of self-condensation of acetyl-CoA. Acetyl-CoA, used as well for formation of mevalonic acid, gives rise to isoprenoids (Baser and Buchbauer, 2010). The other constituents of EOs are derivatives of amino acids other than l-phenylalanine (Dudareva et al., 2006).

### Isoprenoids

Isoprenoids (terpenes) are formed by coupling of isoprene (2-methylbutadiene) units in a pattern called head-to-tail joining, and their structure contains a multiple of five carbon atoms (Mann et al., 1994; Baser and Buchbauer, 2010). Hence, the structural classification of terpenes is based on the number of isoprene units in a molecule. Hemiterpenes are built from one isoprene unit (C5); monoterpenes contain 10 carbons (C10), and sesquiterpenes contain 15 (C15), whereas diterpenes contain 20 (C20). Functional characterization depends on cyclic or linear structure, degrees of unsaturation, or type of substituents (hydrocarbons, alcohols, ethers, oxides, aldehydes, ketones, esters). The term *terpenoids* is restricted to isoprenoids bearing oxygen moiety (Baser and Buchbauer, 2010; Moghaddam and Mehdizadeh, 2017). Some molecules presented in EOs are the degradation products of larger, usually not volatile structures. Norisoprenoids are degradation products resulting from the enzymatic or non-enzymatic cleavage of triterpenoids or tetraterpenoids. Ionones, damascones, or megastigmanes result from degradation of the central part of the chain of carotenoids, whereas irones are the degradation products of triterpenoid iripallidal (Fleischmann and Zorn, 2008; Baser and Buchbauer, 2010).

### Phenylpropanoids

The basic structure in shikimate derivatives is C6–C3 unit of benzene ring (C6) linked usually to three-carbon side chain (C3) at position 1 and oxygenated in the third/fourth/fifth position/s. C3 often possess a carbon–carbon double bond; however, the side chain can also be shortened to one carbon (C1). Phenols or phenol ethers are phenylpropanoids frequently found in EOs (Baser and Buchbauer, 2010; Moghaddam and Mehdizadeh, 2017).

### Derivatives of Polyketides and Lipids

Fatty acid derivatives found in EO are formed in condensation reactions of polyketides, degradation of lipids or cyclization of arachidonic acid (Dudareva et al., 2006). Condensation of polyketides results in formation of phenolic rings, which are oxidized on alternate carbon atoms, either as acids, ketones, phenols, or as one end of a double bond (Baser and Buchbauer, 2010). The array of enzymatic reactions on fatty acids, such as cleavage, oxidation, lactonization, reduction, or elimination, gives rise to short-chain lactones, alcohols, or aldehydes, whereas cyclization or arachidonic acid results in production of prostaglandins and jasmonates (Dudareva et al., 2006; Baser and Buchbauer, 2010).

### Derivatives of Amino Acids Other Than l-Phenylalanine

**Figure 2:**
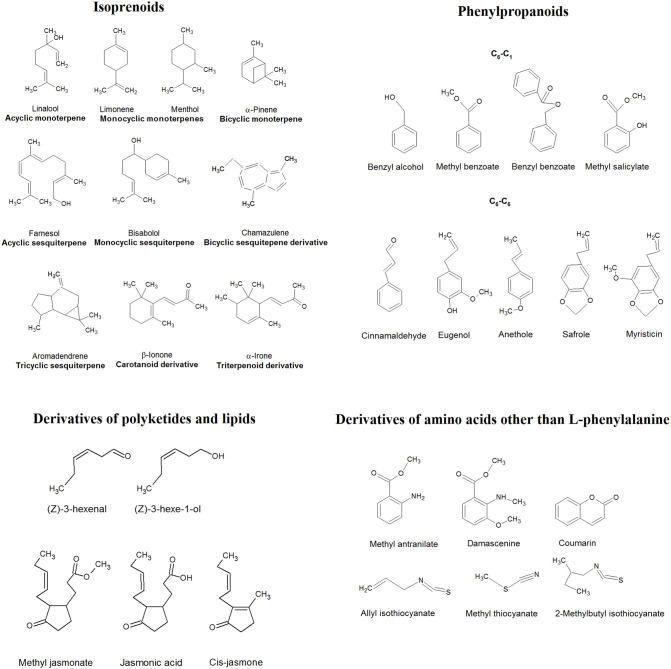
Examples of different groups of EO constituents.

Amino acids, such as alanine, valine, leucine, isoleucine, and methionine, are the precursors of aldehydes, alcohols, esters, acids, and nitrogen- and sulfur-containing constituents of EOs (Dudareva et al., 2006). Sulfur compounds (sulfides, disulfides, trisulfides, sulfoxides, and isothiocyanates), as well as heterocyclic compounds containing nitrogen (indole, methyl anthranilates, pyridines, and pyrazines) or oxygen (lactones, coumarins, and furanoids) in a ring, are rarely found in EOs. These molecules have relatively simple structures and intensive characteristic or pungent odor (Moghaddam and Mehdizadeh, 2017). Some examples of the main groups of EO constituents are presented in Figure 2.

### Physical Characteristics of EOs

The basic characteristic of EOs is their odor. Constituents of EOs are partly in the vapor state due to high vapor pressure at atmospheric pressure and at room temperature (Dhifi et al., 2017). Higher number of carbon atoms in the structure results in decreased volatility. The highest boiling points have monoterpenes; hence, compounds from this class are highly volatile and evaporate quickly. Sesquiterpenes are still sufficiently volatile to be present as EOs; however, diterenes are less frequently found in volatile fractions (Baser and Buchbauer, 2010).

The distillation of plant material usually yields a transparent, colorless, or pale yellow liquid, immiscible with water and having density lower than water. However, some exceptions are known. Solid or semisolid EOs are obtained from orris and guaiac wood or plumeria, respectively. Chamomile gives blue EO; and European valerian, green; and vetiver, brown, whereas cinnamon gives yellow to brownish. Cinnamon, sassafras, and vetiver EOs have density equal to or near one, relative to water (Dhifi et al., 2017; Moghaddam and Mehdizadeh, 2017). Essential oils are soluble in fats, alcohols, and most organic solvents. Their constituents contain asymmetric carbons, what results in optical activity (optical rotation). They are also characterized by refractive index (Moghaddam and Mehdizadeh, 2017). Density, optical rotation, and refractive index are the parameters used to control quality of EOs. Their correct determination is described in standards published by ISO^1^ (ISO/TC 54: ISO 279:1998; ISO 280:1998; ISO 592:1998), and the range of acceptable values is provided for most commercially available EO. Basic physical characteristics of some terpene compounds and EOs are provided in Tables 1, 2, respectively.

**Table 1:** Physical characteristics of selected essential oils constituents.

**Name** **IUPAC Name** **(chemical class)**	**Molecular formula** **Molecular weight**	**Organoleptic** **description**	**Flash point**	**Solubility**	**Density** **(g/cm^**3**^)**	**Index of** **refraction**	**Optical** **rotation**	**Log*P***	**Natural** **occurence**	**Stability**	**References**
Linalool 3,7-dimethylocta-1,6-dien-3-ol (acyclic monoterpene)	C_10_H_18_O 154.25 g/mol	Colorless to pale yellow liquid with floral, spicy, wood odor	160°F	Soluble in alcohol, ether, fixed oils, propylene glycol; insoluble in glycerin	0.870 at 15/4°C	1.4627	−2 to +2°	2.97	Lamiaceae (Origanum, mint, thyme), Lauraceae (laurels), Rutaceae (citrus fruits)	Forced autoxidation after exposure atmospheric oxygen; stable in complex fragrances stored in half-empty bottles, opened every 14 days; only traces of hydroperoxides detected	Baser and Buchbauer, 2010; PubChem, n.d.
α-Pinene 2,6,6-trimethylbicyclo [3.1.1] hept-2-ene (bicyclic monoterpene)	C_10_H_16_ 136.23 g/mol	Clear colorless liquid with a turpentine odor	91°F	Soluble in alcohol, chloroform, ether, glacial acetic acid, fixed oils	0.8592 at 20/4°C	1.4663 at 20°C	+51.14 at 20°C/D	4.4	Very widespread, pine trees; *Salvia officinalis*	Autoxidation after exposure to light	Schrader et al., 2001 Baser and Buchbauer, 2010; PubChem, n.d.
Limonene 1-methyl-4-prop-1-en-2-ylcyclohexene (monocyclic monoterpene)	C_10_H_16_ 136.23 g/mol	Clear to light yellow liquid with pleasant lemon-like odor	97°F	Soluble in 5 volumes alcohol; miscible with benzene, chloroform, ether, carbon disulfide, petroleum ether and oils	0.8402 at 20.85/4°C	1.4723–1.4737 at 20°C/D	+94 to +99° at 25°C/D	4.57	Rutaceae (citrus fruits), pines, junipers	Autoxidation in elevated temperatures to form isoprene. It oxidizes easily in moist air to produce carveol, carvone, and limonene oxide.	Karlberg et al., 1992; PubChem, n.d.
Chamazulene 7-ethyl-1,4-dimethylazulene (degradation product of sesquiterpenoid matricin)	C_14_H_16_ 184.28 g/mol	Blue liquid	278°F	Soluble in alcohols and fixed oils	0.9883 at 20°C	1.584	Not specified	3.97	Chamomile, wormwood and yarrow	Not particularly stable, the deep blue color can change to green and even yellow on aging	Baser and Buchbauer, 2010; Molbase, n.d.; PubChem, n.d.
Eugenol 2-methoxy-4-prop-2-enylphenol (Phenylpropanoid)	C_10_H_12_O_2_ 164.2 g/mol	Clear colorless pale yellow or amber-colored liquid with odor of cloves	212°F	Soluble in all proportions of alcohol, ether, chloroform, or glacial acetic acid.	1.0652 at 20°C	1.5405 at 20°C/D	−1°20′ to −0°49′	2.27	Cloves	Darkens and thickens on exposure to air	PubChem, n.d.
Methyl anthranilate methyl 2-aminobenzoate	C_8_H_9_NO_2_ 151.16 g/mol	Pale yellow liquid with bluish fluorescence and odor of grapes	212°F	Slightly soluble in water; freely soluble in alcohol or ether; soluble in fixed oils, propylene glycol, volatile oils; slightly soluble in mineral oil; insoluble in glycerol	1.168 at 20°C	1.5810 at 25°C/D	Not specified	1.88	Grapes, bergamot, citrus fruits, jasmie	Undergoes direct photolysis under UVC and UVB irradiation	Lanzafame et al., 2017; PubChem, n.d.

**Table 2:** Physical characterization of selected essential oils.

**Name**	**Organoleptic description**	**Solubility**	**Density (g/cm^**3**^)**	**Index of refraction**	**Optical rotation**	**Stability**	**References**
Mentha piperita essential oil	Colorless to pale yellow-greenish liquid with Strong peppermint odor and sweet, balsamic taste, often masked by the distinct cooling effect	Soluble in 70% ethanol at 20°C	0.896–0.908 at 25°C	1.460–1.464 at 20°C/D	−28 to −17° at 20°C	Not specified	PubChem, n.d.
Cinnamon bark essential oil	Dark yellow clear oily liquid with sweet spicy aldehydic aromatic cinnamyl woody resinous honey powdery odor	Soluble in 70% ethanol at 20°C	1.010–1.030 at 25/25°C	1.5730 to 1.5910 at 25°C	−2 to 0 at 20°C	Darkens and thickens on exposure to air	Good Scents Company, n.d.; PubChem, n.d.
Thyme essential oil	Colorless clear liquid with herbal, phenolic odor	Soluble in 70% ethanol at 20°C	0.941 at 25°C	1.506 at 20°C/D	21.0 to +15.0	Not specified	Good Scents Company, n.d.; PubChem, n.d.
Cumin essential oil	Light yellow to brown liquid with strong, somewhat fatty and green odor	Soluble in 80% ethanol at 20°C; Soluble in fixed oils, mineral oil; very soluble in glycerine, propylene glycol, chloroform and ether	0.908–0.958 at 20°C	1.4940–1.5160 at 20°C	+1 to +8 at 20°C/D	Tends to darken on aging; quite sensitive to daylight, air, moisture and metals, as well as alkali.	PubChem, n.d.
Nutmeg essential oil	Colorless or pale yellow liquid with odor and taste of nutmeg	Soluble in fixed oils, mineral oil; slightly soluble in cold alcohol; very soluble in hot alcohol, chloroform, ether; insoluble in glycerine, propylene glycol, and water	0.859–0.924 at 25°C	1.4740–1.4880 at 20°C/D	+10° to +30 at 20°C/D	Not specified	PubChem, n.d.

### Stability of EOs

During release from plant structures (ducts or glands), volatiles become susceptible to temperature, light, oxidation, or hydrolysis. The final composition of EOs depends not only on chemical composition of plant material, but also on plant material processing and storage, distillation processes, and subsequent handling of EOs (Turek and Stintzing, 2013; Moghaddam and Mehdizadeh, 2017). The major factor influencing stability of EOs is the chemical character of their constituents. Compounds containing double bonds are prone to autoxidation because hydrogen atom abstraction results in resonance-stabilized radicals. Polyunsaturated terpenic hydrocarbons can form radicals stabilized by conjugated double-bonds. At the same time, isomerization to tertiary radicals can take place, leading to oxidative deterioration (Turek and Stintzing, 2013). The access to aerial oxygen causes the spontaneous free radical chain reactions, resulting in production of unstable hydroperoxides, which decompose in the presence of light, heat, or upon increasing acidity. Monovalent to polyvalent alcohols, aldehydes, ketones, epoxides, peroxides, acids, or oxygen-bearing polymers are stable secondary oxidation products. Some oxygen-bearing terpenoids are, however, directly converted into oxidized secondary products without hydroperoxides formation (Geier, 2006; Turek and Stintzing, 2013). Because the oxygen present in headspace diffuses into the sample over storage time, the EOs should be kept in completely filled containers or, if possible, should be treated with inert gas to remove remaining air and prevent oxidative reactions (Geier, 2006; Turek et al., 2012). The two other factors strictly correlated with oxidative deterioration of EOs are light and temperature. Light accelerates autoxidation and formation of alkyl radicals, catalyzes intramolecular isomerization reactions or *trans*–*cis* conversions in monoterpenes, and increases the degradation of monoterpenes (Turek and Stintzing, 2013). Heat accelerates chemical reactions and contributes to formation of primary auto-oxidation products—hydroperoxides, which are subsequently decomposed with increasing temperature, resulting in final oxidation products (Turek and Stintzing, 2012, 2013; Turek et al., 2012). Volatiles are thermolabile and susceptible to rearrangement processes at elevated temperatures. Thermal degradation of terpenes is classified into four types of oxidative reactions: cleavage of double bonds, epoxidation, dehydrogenation into aromatic systems, and allylic oxidation into alcohols, ketones, and aldehydes (McGraw et al., 1999). Because oxygen solubility is lower at elevated temperatures, alkyl or hydroxyl radical formation is more pronounced. On the other hand, storage of EOs at low temperatures favors the solubility of oxygen in liquids and results in peroxide formation (Turek and Stintzing, 2013). In complex mixtures, such as EOs, compounds easily undergo oxidation, predominantly isoprenoids, affecting more stable structures initiating their rearrangement and decomposition reactions. In return, phenylpropanoids present as EOs act as antioxidants able to scavenge free radicals and to protect other molecules from deterioration (Turek and Stintzing, 2013). As a result of decomposition processes described above, EOs are losing their quality. The most evident signs of aging are changes in colors, consistency, and odor, and at the last being as unpleasant and often pungent.

General physicochemical features of EOs (complexity and interactions of individual compounds) and their constituents (low molecular weight, presence of different functional groups in the molecule, reactivity, and hydrophobicity) strongly affect the biological activity of EOs.

## Bioavailability of EOs

Essential oils and/or their already extracted individual compounds are well-characterized primarily in the context of their strong antimicrobial (e.g., Burt, 2004; Nazzaro et al., 2013; Semeniuc et al., 2017) and antioxidant activity (e.g., Miguel, 2010). The antimicrobial effects of EOs are connected to their ability to penetrate through bacterial cell wall, where disruption of the bacterial wall induces leakage of ions, reduction of membrane potential, disruption of membrane enzymes, and alterations in structural and functional properties of bacterial cell (Edris, 2007). The EO compounds have strong antioxidant capacity linked to their H-donating properties, ability to inhibit lipid autoxidation, the quenching of singlet oxygen, hydrogen transfer, or electron transfer (e.g., Grassmann, 2005). Besides these effects, EOs are much studied for their anticancer (e.g., Bayala et al., 2014), anti-inflammatory (Sá et al., 2014), anxiolytic (De Sousa, 2012), analgesic-like (De Sousa, 2011), antinociceptive (Lenardão et al., 2016), and antiaging and neuroprotective effects (Ayaz et al., 2017). Complex and various bioactive roles of EOs and their compounds were summarized in several comprehensive reports (e.g., Edris, 2007; Bakkali et al., 2008; Raut and Karuppayil, 2014; Sarkic and Stappen, 2018). However, the behavior of EOs and their individual compounds in human body upon administration is far less revealed.

The term *bioavailability* refers to pharmacokinetic properties of the drug after reaching the systemic circulation allowing its actions at the target sites (Stahl et al., 2002). Bioavailability, in fact, includes two interconnected subsets: the bioaccessibility and the bioactivity. The concept of bioaccessibility is defined as the quantity or fraction that is released from the food matrix in the gastrointestinal tract being available for absorption (Thakur et al., 2020). It also describes the availability of compound for assimilation after digestive transformations, the absorption, and, finally, the presystemic intestinal and hepatic metabolism (Cardoso et al., 2015). The concept of bioactivity includes the processes of drug entering into systemic circulation, its transportation to the target site, and interactions with different biomolecules, resulting in expression of various metabolic and physiological effects (Wood, 2005; Carbonell-Capella et al., 2014). Bioavailability is the key aspect for assessment of drug absorption applied via different administration routes (Maderuelo et al., 2019).

### Approaches in Bioavailability Studies

Bioavailability and bioaccessibility of plant metabolites, including EOs and their individual terpene compounds, are studied by different *in vivo* and *in vitro* methods. *In vitro* digestion models are commonly used for bioaccessibility estimation. The most of these methods simulate the conditions of gastrointestinal (GI) system by adjusting the pH and introduction of particular digestive enzymes (e.g., salivary amylase, pepsin, gastric lipase, trypsin, chymotripsin, pancreatic lipase, etc.), bile salts, and, sometimes involving fermentation reactions to reproduce the colon performance (Jones et al., 2019). Recent *in vitro* bioaccessibility/bioavailability studies include cell models, primarily Caco-2 cells isolated from the human colorectal adenocarcinoma, where absorbed target compound is collected on the basolateral side of the monolayer model cells (Jones et al., 2019; Thakur et al., 2020). Bioavailability studies have been also performed by *in vivo* animal and clinical studies.

As for the other drugs, bioavailability of EOs and their compounds represents the concentration threshold reaching the blood circulation system and includes digestion (in case of oral administration), absorption, transformation (metabolism), tissue distribution, and bioactive performance at the target sites (Carbonell-Capella et al., 2014).

Bioavailability comprises pharmacodynamic and pharmacokinetic performances of bioactive compounds. Pharmacodynamics of EOs includes effects of their individual compounds on human and animal biochemical and physiological processes, i.e., should include the monitoring of biological activity at target organs, tissues, and cells. Independently of low selectivity of EOs, their bioactive effects are expressed after reaching the blood circulation.

Pharmacokinetics of EOs in fact reflects the destiny of each individual compound from the intake toward final elimination from the body, referring different processes of bioaccessibility and bioavailability.

The primary intake routes of EOs are skin application, inhalation, and oral intake.

### Bioavailability of EOs in Relation of Administration Routes and EO Absorption

Various factors affect the bioavailability of EOs, such as physiochemical, biochemical, and physiological interactions. The comprehension of bioavailability of EOs includes the monitoring of successive phases of their absorption, distribution, and excretion in the human body. There is limited information on their behavior and fate in human, and most studies are performed either *in vitro* (e.g., Volić et al., 2018) or on animal models (e.g., Michiels et al., 2008; Zhang Y. et al., 2014). It is assumed that bioavailability of EOs is maximal (100%) by intravenous administration and decreases upon other administration routes. However, the bioavailability of EO constituents orally administrated might be very high, as reported for 1,8-cineole with bioavailability rate of 95.6% (Zimmermann et al., 1995).

Nevertheless, recent reports confirm that most EOs are rapidly absorbed under dermal, oral, or pulmonary administration. To assume the bioavailability and especially the bioactivity of EOs, it is necessary to know how and in which amount they could enter the blood circulation and how they are distributed within the body, all reflecting the safety issues of EOs (Tisserand and Young, 2014).

#### Dermal Administration

The skin consists of outer and deeper dermis, where the stratum corneum, the outer epidermis layer, is the first physical barrier for penetration of external chemicals (Godin and Touitou, 2007). It is assumed that there are three possible skin penetration pathways: the intercellular (between the skin cells), the transcellular (through the cells), and the route through the hair follicles, bypassing the stratum corneum (Williams and Barry, 2012). It is already known that most of EOs' constituents penetrate from skin surface and through the stratum corneum, toward the dermis, and finally into the blood circulation (Tisserand and Young, 2014). Regarding lipophilic feature of EOs, the high percutaneous absorption rates should be considered in risk assessments in systemic toxicity.

Hydrophilic drugs are better absorbed in combination with terpenes containing polar functional groups, and similarly, the absorption of lipophilic compounds is improved in the presence of hydrocarbon terpenes (Godwin and Michniak, 1999). In general, lipophilic drugs pass the dermal barrier more intensively than the hydrophilic substances (Wester and Maibach, 2000). Terpene compounds are also used for the improvement of the transdermal drug delivery (Aqil et al., 2007), mainly due to high percutaneous enhancement ability and low cutaneous irritancy at concentrations of <5% (Nokhodchi et al., 2007). In addition, EOs are known for their positive effects on skin and prevention and healing of some dermatological disorders (e.g., Sarkic and Stappen, 2018). There are recent applications of EOs encapsulated by liposomes in cosmetics (Sherry et al., 2013).

#### Respiratory Administration

Inhaled substances are transported via trachea into the bronchi and then to bronchioles and finally toward lung alveoli, which are very efficient in transporting small molecules, such as terpenes, into the blood circulation (Tisserand and Young, 2014). Beneficial effects of EOs on respiratory system by inhalation are well-accepted (e.g., Maddocks-Jennings and Wilkinson, 2004).

#### Rectal and Vaginal Administration

Rectal suppositories are used in the case when high systemic concentrations are desired for bioactivity in the colon. However, dosage and concentrations should be carefully adjusted because of the high sensitivity of rectal mucous membrane for EOs and possible irritations (Tisserand and Young, 2014). Similar finding refers to the vaginal application of EOs, where additional emulsification is required. It might be expected that encapsulation of EOs with adequate carriers for these applications will be developed in future experiments.

#### Oral Administration

Oral administration of free EOs is generally performed by dilution with milk, soy milk, olive oil (Bilia et al., 2014), or other vegetable oils. However, oral intake is the most common way in application of encapsulated EOs, especially in case of food supplements and functional foods. Entrapping of EOs and/or their already extracted individual bioactive compounds (e.g., menthol, chamazulene, thymol, carvacrol, limonene, 1,8-cineole, etc.) enables targeting and controlled release of EOs, protects EOs from degradation and losses, and allows masking of unpleasant taste and odor (e.g., Bilia et al., 2014; Asbahani et al., 2015; Dajić Stevanović et al., 2018) via oral intake. Bioavailability of the matrix and EO components is complex and should be considered from the aspect of stability of microencapsulated or nanoencapsulated carriers within the GI tract, as well as enhanced bioavailability and, particularly, the systemic activity of a drug at the action site.

Gastrointestinal tract is tube-like of interconnected compartments containing associated organs, such as liver, pancreas, and gallbladder. The upper GI tract is composed of the oral cavity and salivary glands, the esophagus, stomach, and small intestine (duodenum, jejunum, and ileum), whereas the lower GI tract is represented by the large intestine (cecum, colon, and rectum) (Treuting et al., 2018). The GI tract is enveloped by four concentric layers: the innermost mucous layer, with the presence of mucus and HCl secreting glands; a submucosal layer; and the outer muscular and the outermost serous layers (Maderuelo et al., 2019). The principal risk of administering of EOs or their constituents to any part of the GI tract is irritation and inflammation of the mucous membrane whose irritation is highly concentration-dependent (Tisserand and Young, 2014). Nevertheless, many reports highlighted the beneficial effects of EOs and their compounds on gastric mucosa, where gastroprotective activity was associated with different mechanisms, such as activation of a2-receptors, increased HSP-70, VIP, and PGE_2_ expression (heat shock protein, vasoactive intestinal peptide, and prostaglandin, respectively), and gastric SH group bioavailability (Rozza and Pellizzon, 2013). According to Fernandes et al. (2012), the lemongrass EO reduces gastric damage due to mechanisms involving endogenous prostaglandins, while gastroprotective action of orange EO and the limonene is related to an increase in the gastric mucus production (Moraes et al., 2009). Therefore, in some cases, it is reasonable to ensure the complete release of EO from coating material in the stomach due to positive effects on bioactive compounds on the gastric mucosa.

Besides responsibility for digestion due to low gastric pH and enzymatic activity of pepsin, the stomach allows absorption of water and some substances, including some particular terpenes (which should be less lipophilic, i.e., those containing polar groups, such as oxygenated monoterpenes, such as linalool, geraniol, neral, thymol, citronellol, etc.). In experiments performed through *in vitro* conditions and *in vivo* on piglets model, it was shown that thymol, eugenol, carvacrol, and *trans*-cinnamaldehyde were weakly degraded in the proximal segments of the GI piglet tract under *in vitro* conditions. *In vivo* studies showed that these EO constituents were almost completely absorbed in the upper GI tract, by the stomach and the proximal small intestine (Michiels et al., 2008). Digestive enzymes can break down some types of EO constituents; for example, esters may be hydrolyzed in the stomach (Tisserand and Young, 2014).

The intestine as the main absorption site is characterized by high absorption area due to the presence of villi and microvilli. The absorption rate of EOs constituents highly depends on some factors, such as molecular weight, lipophilicity, solubility, and polarity (Esfanjani et al., 2018). Therefore, different terpenes are absorbed at different rates and by different parts of the GI tract. It was shown that the upper GI tract is not responsible for absorption of 1,8-cineole (Kohlert et al., 2000), in contrary to the fast absorption of thymol (Kohlert et al., 2002), confirming former results with ileostomy patients (Somerville et al., 1984). The rapid absorption of terpenes was confirmed by detection of 1,8-cineole and α-pinene in plasma just after 30 min after oral application (Zimmermann et al., 1995). Mixing of EO components with pancreatic juice and bile salts in duodenum would result in their better solubilization and related rapid absorption (Michiels et al., 2008). The fast absorption of EO compounds is attributed to their small size and lipophilic nature (Kohlert et al., 2002). It was postulated that most of the substances are absorbed in the GI tract by a passive diffusion upon transcellular and paracellular absorption pathways, although some drugs have to be absorbed by carrier-mediated transport according to their responses to membrane permeability (Ho, 2011). For intestine delivery and absorption, there is need to ensure stability of encapsulate and, especially of bioactive material, within the gastric stage.

In general, the bioavailability of drugs is highly dependent on fluctuations of GI pH values. The intraluminal pH varies along the GI tract, from very acidic in the stomach (lowest values in digesting phase are 1–2 and about 4–5 during the resting phase) to neutral in the large intestine; pH values increase up to 5.5 in the upper small intestine, reach the maximum of 7.5 in the ileocolonic region, then decrease to 5.7 in the cecum, and increase again to pH of ~6.7 in the rectum (Maurer et al., 2015). It could be assumed that GI absorption of different EOs constituents will follow pH partition theory, via passive transport of lipophilic, unionized compounds through biological membranes (Maderuelo et al., 2019). The pH of the stomach as being acidic favors the absorption of weak acids, whereas the pH of the small bowel, being closer to neutrality, facilitates the absorption of weak bases, as stated by Abuhelwa et al. (2017). It is thought that alcohols (and hence the terpene alcohols) act as both weak acids and weak bases, depending on factors, such as electronegativity, inductive and resonance effects and polarizability, as well as salvation as an extrinsic factor (Roberts and Caserio, 1977). In that sense, it could be expected that terpene alcohols, such as citral, linalool, geraniol, 1,8-cineole, and so on, might behave either as acids or as bases, depending on microenvironmental GIT conditions, in the first pH, which will much influence their absorption site. On the other hand, terpenes with a carboxylic group (e.g., carnosic acid, abietic acid) and phenolic terpenes (e.g., thymol, anethol, carvacrol) usually behave as weak acids and thus might be absorbed in the stomach, at least partially. Finally, N-containing terpenes, mainly derivatives of amino acids other than l-phenylalanine and aminated terpenes, might act as a weak bases as will be more readily absorbed in the intestine. However, the absorption mechanisms are much more complex and cannot simply follow the pH partition theory, due to different intermolecule interactions, changes in molecule structure under different conditions of GI tract, and special features of each individual body. Therefore, it is pretty difficult to predict the bioavailability of particular EOs constituent only upon its physicochemical properties, such as molecular mass, functional group, stability, solubility, responses to pH, and so on. These data are insufficiently informative for speculations on interactions of EOs with a range of biomolecules in such complex biological systems, as tissues and organs of a human body.

### Metabolism, Distribution, and Excretion

To enhance bioavailability, the bioactive compound should exhibit sufficient absorption and low (renal) clearance, i.e., excretion ability. As soon as an EO component comes in the bloodstream, the body begins to modify it, i.e., to break it into the smaller and more polar molecules for easier kidney filtration and elimination (Djilani and Dicko, 2012).

Liver metabolism includes transformation processes of oxidation and hydroxylation, as well as adding of some polar accessories, known as phase I (metabolism via the cytochrome P450 path) and phase II (glucuronidation, sulfation, and glutathione conjugation), respectively. Metabolic fate is highly dependent on the chemical nature of EOs and their individual compounds. Essential oil metabolites of phase II were found in human and animals, in form of glucoronides and sulfates, whereas excreted metabolites are mainly glycine and glucuronic acid or are exhaled as CO_2_ (Kohlert et al., 2002). Terpenes are distributed from blood circulation to other tissues. Because of high clearance and short elimination half-life, their accumulation is doubtful. It was shown that, after oral administration of menthol, 35% of the original amount of the compound was excreted renally as menthol glucuronide, the major biliary metabolite able to enter enterohepatic circulation (e.g., Kohlert et al., 2000; Grigoleit and Grigoleit, 2005). The similar was reported for thymol, carvacrol, limonene, and eugenol. After oral intake of EOs, sulfate and glucuronide metabolites have been found in urine and in plasma, respectively (Guénette et al., 2007; Michiels et al., 2008).

Lipophilic substances, such as EO components, are able to penetrate the blood–brain barrier and to interact with various brain receptors, such as γ-aminobutyric acid and glutamate receptors (Tisserand and Young, 2014). Monoterpenes and sesquiterpenes would be expected to spend a short time in the bloodstream before being redistributed first to muscle and then over a longer period to fat (Tisserand and Young, 2014). However, it is known that EO compounds are easily absorbed, and only a small portion of the EO remains unchanged (e.g., Kohlert et al., 2000; Djilani and Dicko, 2012), independently on administration route. The fast metabolism and short half-life of active compounds of EOs ensure minimum risk of their accumulation in body tissues (Kohlert et al., 2002).

Characteristic and distinctive properties of EOs and their constituents relating their physicochemical characteristics, bioavailability, and especially biological effects at the target site are a crucial point for the determination of appropriate carrier system and related encapsulation technique.

## Needs for Microencapsulation of EOs: Encapsulation Technologies and Selection of Carrier Systems

Microencapsulation of active compounds, including EOs, has two functions: (1) to enhance the oxidative stability, thermostability, photostability, shelf life, and biological activity and (2) to ensure their target delivery (Gallardo et al., 2013; Yang and McClements, 2013; Martins et al., 2014; Xiao et al., 2014; Yang et al., 2015). Specifically, encapsulation of EOs controls the volatility, sensory (mainly odor and taste), and release properties of EOs and ensures the prolonged chemical stability and biological activity under storage conditions (e.g., Bilia et al., 2014).

Several reviews pointed out that the bioactivity of EOs is improved by encapsulation (e.g., van Vuuren et al., 2010; Bilia et al., 2014; Dima et al., 2014; Asbahani et al., 2015; Li et al., 2015; Pandit et al., 2016; Maderuelo et al., 2019). For example, encapsulated peppermint oil in starch-based emulsions exhibited increased bioavailability and stability characteristics and enhanced activity against *Listeria monocytogenes* and *Staphylococcus aureus* compared to free EO (Liang et al., 2012). It was shown that the antibacterial activity of EOs after their nanoencapsulation very often succeeded to surpass the efficiency of current antibiotic (Zaman et al., 2017).

The enhancement of the biological activity of encapsulated EO could be primarily attributed to better stability and reduced exposure to degradation processes by entrapping. However, it was hypothesized that in some cases such effects are a result of synergistic effects by interactions of EOs with some carrier materials, such as cashew gum or chitosan (e.g., Pandit et al., 2016). Finally, the encapsulation in nanometric particles *per se* contributes to better cellular absorption mechanisms and the bioefficacy (Bilia et al., 2014).

**Figure 3:**
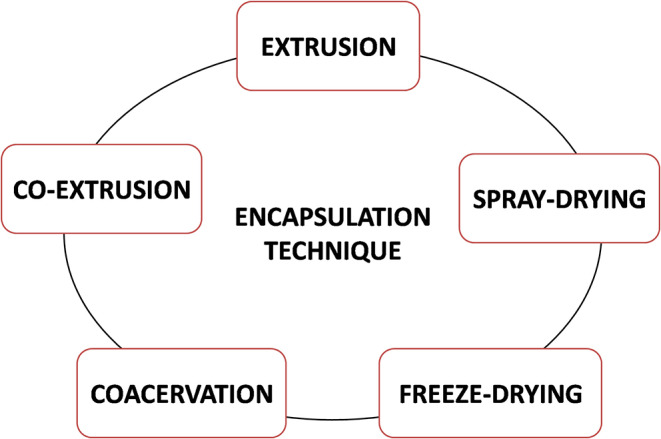
Different techniques for EO encapsulation.

The stability, release kinetics, and related bioavailability and bioactivity of encapsulated material are highly dependent on encapsulation technology. Several encapsulation techniques, such as spray drying, extrusion, coacervation, emulsification, and so on, are used today for encapsulation of EOs (Figure 3), depending on main features of core material and the carrier, as well as application of encapsulated material. Spray drying is a rapid, continuous, relatively low-cost production operation and easy production scale-up. Biomolecules applied as carriers for the spray-drying technique are maltodextrins, starch, gum Arabic, and chitosan (Ersus and Yurdagel, 2007; Kausadikar et al., 2015). Disadvantages of spray-drying technique in general are as follows: (1) non-uniform particles size and shape, (2) tendency of particles to aggregate, (3) carrier material solubility in water at an acceptable level, and (4) short-time exposure to high temperatures.

Coacervation could be suitable technique for covering EO droplets by single shell (simple coacervation) or two-layer shell (complex coacervation). Shell material should ensure (1) rigidity of oil carrier, (2) thermal stability, and (3) chemical stability under gastric condition and (4) should be dissolved in intestine fluid. Two-layer shell consists of inner layer near the oil droplets and outer layer. Inner layer can be made from amphiphilic materials, such as proteins or some surfactants (Torcello-Gomez et al., 2011). Outer layer usually consists of polysaccharide hydrogels suitable to ensure mechanical stability of oil carriers and satisfy proposed process conditions. Extrusion is the technique that can be used in combination with coacervation. This technique is suitable for preparing (1) polysaccharide monophase and multiphase hydrogel matrices, and (2) polysaccharide–protein hydrogel blends in the form of microbeads (Nedović et al., 2011; Volić et al., 2018; Obradovic et al., 2019). The advantages of extrusion are (1) the chemical stability of beads under storage conditions and gastric conditions, (2) the mechanical stability of the beads, and (3) the possibility of encapsulation of hydrophobic or hydrophilic active compounds. The disadvantages of this technique could be (1) low production rate and (2) scale-up difficulties (Gouin, 2004). Another frequently used technique is emulsification. Emulsions, such as vegetable oils with addition of proteins and emulsifiers represent a good choice as carriers for encapsulation of hydrophobic active compounds. Emulsification technique is suitable for preparing the small-sized particles (10 μm−1 mm) compared with the extrusion technique. However, processing costs seem to be higher than for extrusion.

The choice of carrier material for oral administration depends on (1) the surface activity of active compound, (2) processing conditions, (3) storage conditions, and (4) cost and scale of production. In the context of target delivery of carriers to lower intestine, they should keep their integrity under gastric conditions during retardation time. In order to optimize carrier's performance, it is necessary to consider (1) the interactions of pepsin with the main constituents of carrier's matrix at molecular level and (2) carrier structure at supramolecular level.

Carriers, often used in the literature, could be classified into two groups: (1) carriers made by natural macromolecules and (2) lipid-based carriers. Carriers made by natural macromolecules can be divided in few groups: (1) monophase polysaccharide hydrogels, (2) multiphase polysaccharide hydrogels in the form of blends or multilayer microbeads, and (3) polysaccharide–protein hydrogels in the form of blends, (4) lipid-based carriers, such as some vegetable oils and liposomes, and (5) lipid–protein carriers. Various polysaccharide ionic hydrogels, such as Ca–alginate (Chan, 2011), alginate–cashew gum (de Oliveira et al., 2014), cashew gum–inulin (de Barros Fernandes et al., 2016), alginate–xanthan gum (Zhang S. et al., 2014), xanthan gum–pectin (Qiu et al., 2015), alginate–pectin (Wang et al., 2013), and alginate–chitosan (Xu et al., 2007), have been used as the carrier's matrix primarily for the entrapment of hydrophilic active compounds, such as some types of polyphenols. Polysaccharide–protein hydrogels are suitable for the entrapment of hydrophobic active compounds, such as EOs and some types of polyphenols because of the amphiphilic properties of proteins. Frequently used proteins blended with alginate, pectin, and xanthan are lupin (Piornos et al., 2017), soybean lecithin (Torcello-Gomez et al., 2011), gliadin (Qiu et al., 2015), whey proteins (Zhang et al., 2016), gelatin (Roy et al., 2009), and many others. Liposomes are suitable for the encapsulation of hydrophilic and hydrophobic active compounds (Akbarzadeh et al., 2013). Vegetable oils reach by long-chain triglycerides are a good choice for the encapsulation of EO because of their resistance to the action of pepsin (Yara-Varon et al., 2017).

### Polysaccharide-Based Carriers

Polysaccharides, such as alginate, chitosan, and maltodextrin, are widely used in the form of physical or chemical hydrogels for encapsulation of EOs (Ravichandran et al., 2014; Gomez-Mascaraque et al., 2015; Pasukamonset et al., 2016). Alginate is a common name for a whole family of natural, water-soluble, linear macromolecules of high molecular mass (between 32,000 and 400,000 g/mol) primarily extracted from brown algae species. The distribution of mannuronic acid and guluronic acid units in alginate chain containing blocks of G-G, M-M, and M-G residues [where G is α-l-gluronic acid, and M is β-d-mannuronic acid Milivojevic et al., 2015], as well as their ratio (M/G), depends on the natural source of alginate (type of algae, season, location etc.) and predominately determine their physical and chemical properties. Chitosan is composed mainly of (1, 4) linked 2-amino-2-deoxy-d-glucan. Similarly as alginate, the chitosan chains also behave as semiflexible chains. Anion-like alginate chains spontaneously form gel with cation-like chitosan chains at pH 7.0 with various rheological behavior, depending on the alginate-to-chitosan ratio. Maltodextrin is extracted from starch by partial hydrolysis. This type of polysaccharide consists of d-glucose units connected in chains of variable length.

Ca–alginate has been proposed in order to improve bioavailability, thermal stability, and biological activity of active compounds under simulated GI conditions (Cho et al., 2014; Pasukamonset et al., 2016). However, undesirable leakage of EOs could be resulted by weak interactions between active compounds and hydrogel matrix. In order to improve the carriers performances, Ca–alginate beads could be coated with chitosan. The additional stability of carriers is provided by formation of alginate–chitosan complexes (Popa et al., 2000; Anbinder et al., 2011). Devi and Maji (2009) used chitosan–carrageenan carriers to encapsulate neem EO. Neem seed oil is a commercialized product derived from fruits of the neem tree, also named margosa oil. Microencapsulation of pimento EO in chitosan and k-carrageenan ensures antimicrobial activity against various bacteria, such as *Candida utilis, Bacillus cereus*, and *Bacillus subtilis* (Dima et al., 2014). Dong et al. (2011) used gum Arabic as carriers for microencapsulation of peppermint EO. Prolonged release of peppermint EO (and its major compounds, mainly menthol and isomenthol) is ensured by encapsulation (Sarkar et al., 2013).

Maltodextrin is a hydrolyzed starch commonly used for microencapsulation of EOs in combination with surface active biopolymers, such as gum Arabic (Fernandes et al., 2008; Bule et al., 2010; Kausadikar et al., 2015), modified starches (Bule et al., 2010), and proteins (Hogan et al., 2003; Bae and Lee, 2008) in order to ensure an effective encapsulation by spray drying process. It is in accordance with the fact that maltodextrin provides good thermal stability and protection against oxidation. However, this polysaccharide ensures low emulsifying capacity. Consequently, it is desirable to mix maltodextrin with surface-active biopolymers in order to advance the volatile retention of bioactive compounds during the drying process (Ersus and Yurdagel, 2007; Fang and Bhandari, 2010; Paz et al., 2010; Mahadivi et al., 2016; Tolun et al., 2016). Composite made by gum Arabic, modified starch, and maltodextrin has been used for microencapsulation of cardamom EO in order to increase the stability of components, such as 1,8-cineole and α-terpinyl acetate (Krishnan et al., 2005). Kanakdande et al. (2007) used similar carriers for microencapsulation of cumin oleoresin. They reported that cumin volatile EO consists of terpenes (such as β-pinene, *p*-cymene, and γ-terpinene), aldehydes (cuminaldehyde, 1,3-pmentha, and 3-*p*-menthen-7-al), and terpene alcohol. Oxygen barrier properties of maltodextrin depend on the dextrose equivalent (DE). Carriers with higher DE ensure intensive interactions between active compounds and matrix, which ensure higher encapsulation efficiencies. These carriers are less permeable to oxygen.

Maltodextrin with high DE has been successfully utilized in the encapsulation of lemon EO, orange peel EO, cardamom EO, and ginger EO to protect it from oxidation (Touré et al., 2011; Simon-Brown et al., 2016). Recently, it was shown that maltodextrin as carrier material has also an ability to provide protection of the polyphenol compounds against enzyme actions in simulated GI conditions (Romano et al., 2017). The xanthan gum, an extracellular microbial polysaccharide, has been used as a carrier for antioxidants and phenolic compounds (Da Rosa et al., 2013; Rutz et al., 2013). Xanthan gum has potential to be used as a carrier material in combination with maltodextrin and chitosan in order to ensure strong electrostatic interactions, between the amino groups of chitosan (polycation) and the carboxylic groups of xanthan (polyanion). This type of blend carriers has demonstrated improvement in the controlled release rate of encapsulated ingredient (Martínez-Ruvalcaba et al., 2007; Da Rosa et al., 2013). It was also reported that the biopolymer complexes of xanthan gum and whey proteins have emulsifying power and also the positive effect on the control release of water-soluble nutraceuticals in the delivery systems type of W/O/W double emulsions (Benichou et al., 2007; Prichapan and Klinkesorn, 2014).

### Protein-Based Carriers

Proteins as natural food-grade polymers were used: (1) alone and (2) in combination with polysaccharide hydrogel as carrier materials for microencapsulation of many EOs, because of their binding hydrophobic interactions and hydrogen bonding attraction between molecules (Zou et al., 2012; Haratifar and Corredig, 2014; Chuacharoen and Sabliov, 2016). Protein interchain and intrachain self-cross-linking is prerequisite in formation of protein carrier matrix. This crosslinking could be induced in some cases by heat treatment or by changing the pH of a solution (Shpigelman et al., 2010; Tavares et al., 2014). Protein-based carriers, such as gelatin, casein, whey proteins, and soy proteins, have been mostly used for encapsulation of thermosensitive, hydrophobic bioactive compounds (Pool et al., 2013; Xue et al., 2014; Jia et al., 2016). Encapsulation efficiency of these carriers depends on binding affinity of the polyphenols and EOs to protein matrix (Livney, 2010). Release property of bioactive compounds from the protein-based carriers depends on pH conditions. Pronounced swelling of protein-based carriers obtained at neutral pH could induce the undesirable leakage of bioactive compounds (Kimpel and Schmitt, 2015; Liu et al., 2015). Important disadvantage of this type of carriers is significant disintegration under gastric condition caused by pepsin attack (Kumar et al., 2016). Sutaphanit and Chitprasert (2014) used gelatin for microencapsulation of basil EO. Its main components include methyl eugenol (42.58%) followed by caryophyllene (26.88%) and eugenol (10.66%).

Compared to protein-based carriers, combination of protein–polysaccharide carriers can improve mechanical and release properties of the delivery systems and prevent the enzymatic degradation of the proteins in gastric condition (Diaz-Bandera et al., 2013; Jia et al., 2016). In addition, many studies have shown that globular proteins, as well as whey proteins hydrolysates, have antioxidant activity. They can reduce the undesirable oxidation of EOs and provide better oxidative stability of the carriers (Dryakova et al., 2010; Carneiro et al., 2013). The protein–polysaccharide carriers are excellent systems for encapsulation of EOs in order to (1) stabilize these active compounds and (2) protect them from chemical degradation (Turasan et al., 2015; Campelo et al., 2017). Oregano EO emulsion was stabilized by Tween 80 and encapsulated in various types of microcarriers, such as milk powder and whey protein particles, rice starch particles and inulin, and gelatin–sucrose composite (Beirãao da Costa et al., 2012).

Advantage of polysaccharide carriers is in nutrition quality, easy preparation procedure, and low cost. The disadvantage is related to low encapsulation efficiency, loading capacity, and release efficiency in small intestine (de Oliveira et al., 2014). Addition of protein clusters improves their thermal and mechanical stabilities and nutrition quality. The disadvantage of polysaccharide/protein carriers for encapsulation of EOs is also related to low encapsulation efficiency, loading capacity, and release efficiency in small intestine (Dajić Stevanović et al., 2018; Volić et al., 2018).

### Lipid-Based Carriers

Lipid-based carriers made by vegetable oils have been widely used for encapsulation of EOs (Bilia et al., 2014). The main advantages of these carriers are (1) good encapsulation efficiency, (2) stability of active compounds under storage conditions and under gastric condition, and (3) thermal stability (Campos et al., 2014). Vegetable oils represent a mixture of triglycerides (major components) and minor components (<5%), such as glycerolipids, such as monoglycerides and diglycerides, phospholipids, and non-glycerolipids, including sterols, tocopherols/tocotrienols, free fatty acids, vitamins, pigments, proteins, phenolic compounds, water, and so on (Yara-Varon et al., 2017).

Other types of lipid-based particles are liposomes and solid lipid carriers. This type of the carriers can be used for encapsulation of hydrophobic, hydrophilic, and amphiphilic molecules (Yoshida et al., 2010). The thin film hydration, freeze–thaw, sonication, and reverse-phase evaporation were mostly used methods for their preparation and encapsulation of EOs. The disadvantages of liposomes are: (1) complex and expensive preparation procedure and (2) reduced stability under storage conditions that could restrict their applications (Akbarzadeh et al., 2013). Solid lipid nanoparticles and nanostructured lipid carriers were used as nanocarriers for the delivering of polyphenol-type catechins (EGCG) (Shi et al., 2013).

Lipid carriers can be combined with proteins in order to improve their chemical and mechanical stabilities, as well as encapsulation efficiency. Carriers made by mixing of proteins and lipid components (β-lactoglobulin-medium chain triglyceride) have been successfully used for the encapsulation of polyphenols (Pool et al., 2013).

Advantages of lipid carriers are higher encapsulation efficiency, loading capacity, and release efficiency in small intestine in comparison with polysaccharide/protein carriers. However, the disadvantage is related to low mechanical and thermal stabilities, which significantly reduce their usefulness, as well as complex preparation procedure, and higher cost in comparison with polysaccharide/protein carriers (Akbarzadeh et al., 2013).

**Figure 4:**
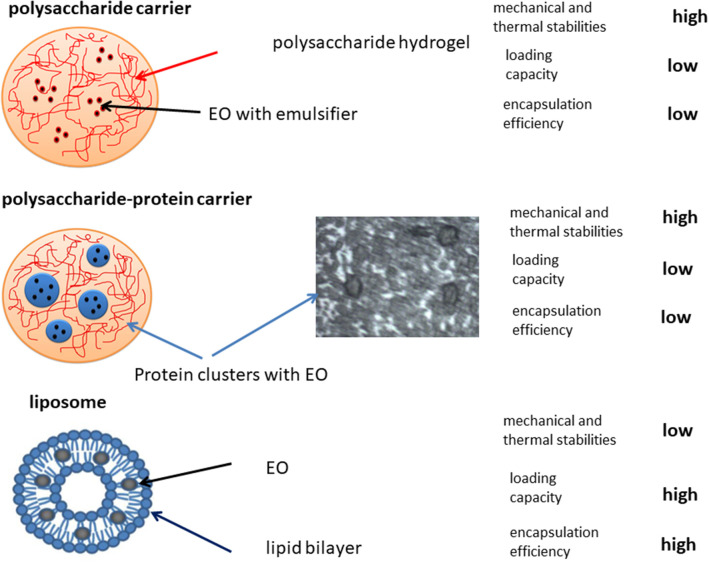
Challenges in carrier selection for EO encapsulation.

Examples of combined carriers for encapsulation of EOs are provided in Table 3, whereas main challenges in choice of carrier systems for EO encapsulation are illustrated in Figure 4.

**Table 3:** Examples for mixed carriers and encapsulation techniques for essential oils.

**Essential oil source**	**Carrier**	**Encapsulation technique**	**References**
Avocado	Whey protein and maltodextrin	Spay-drying	Bae and Lee, 2008
Canola	Alginate, high methoxyl (HM) pectin-enhanced alginate	Coextrusion	Wang et al., 2013
Rosemary	Whey protein concentrate and maltodextrin	Freeze-drying	Turasan et al., 2015
Sweet orange	Soybean protein isolate, gum Arabic	Complex coacervation	Xiao et al., 2014
Holy basil	Gelatin, sodium alginate	Simple coacervation	Sutaphanit and Chitprasert, 2014
Camphor	Gelatin, gum Arabic	Complex coacervation	Xiao et al., 2014
Mint	Guar gum hydrolyzate, gum Arabic	Spay-drying	Sarkar et al., 2013
Thyme	Alginate, soy protein isolate	Electrostatic extrusion	Volić et al., 2018
Linseed	Alginate, lupin protein	Extrusion	Piornos et al., 2017
Sweet peppermint	Gelatin, gum Arabic	Complex coacervation	Dong et al., 2011
Pimento	Chitosan, k-carrageenan	Complex coacervation	Dima et al., 2014
*Lippia sidoides*	Maltodextrin, gum Arabic	Spray-drying	Fernandes et al., 2008
Lavandin	Soybean lecithin, and cholesterol	Thin-film hydration	Varona et al., 2011
Blue gum	Diastearoyl phosphatidylcholine, chitosan	Reverse phase evaporation	van Vuuren et al., 2010
*Eucalyptus camaldulensis*	Soya lecithin and cholesterol	Freeze–thaw	Moghimipour et al., 2012
Rosemary	Cholesterol, phosphatidyl choline	Thin-film hydration; sonication	Arabi et al., 2017
Brazilian cherry	Hydrogenated soy lecithin	Thin-film hydration	Yoshida et al., 2010

### Chemical Stability of Carriers Under Gastric Conditions

Chemical stability of carrier matrices under gastric condition is considered at molecular level and supramolecular level in order to improve their resistance to the attack of pepsin. Affinity and activity of pepsin to the various components of carrier's matrix, such as polysaccharides and proteins under gastric condition, are considered at molecular level in the context of various physical and chemical factors. The structural changes of carrier's matrix represent the cumulative effects of pepsin interactions with the carrier's components and could lead to undesirable carrier weakening and the leakage of active compounds. Deeper understanding of these structural changes is necessary in order to improve the carrier's performances.

### Affinity and Activity of Pepsin

Pepsin is the one of the aspartic protease enzymes. All members of this class of enzymes have two aspartic acid residues within their structure that act as the active site. Pepsin could not form chemical bonds with biopolymers. The mechanism of pepsin action is related to the ability of the two aspartic acids at the reaction site to simultaneously act as both an acid and a base.

Affinity and activity of pepsin to biopolymers, such as polysaccharides and proteins under gastric conditions depend on chemical and physical factors. The physical factors of biopolymers are (1) isoelectric point; (2) the surface activity of biopolymer; (3) the flexibility of chains; (4) the conformation of chains under gastric condition, which is related to its hydrophobic/hydrophilic behavior; and (5) chain length. Chemical factors are related to (1) possible pepsin attack to biopolymers and (2) possible pepsin inactivation caused by the presence of biopolymers. Pepsin attack is directed to glycoside bonds (characteristic for polysaccharides) and the peptide bonds of aromatic amino acids, such as tryptophan, tyrosine, phenylalanine, and glutamate (characteristic for proteins). Pepsin inactivation is induced primarily by carboxyl groups and hydroxyl groups of biopolymers.

Polysaccharides rich by carboxyl groups, such as alginate, maltodextrin, and pectin, can inactivate pepsin (Strugala et al., 2005). At the same time, pepsin can hydrolyze these polysaccharide chains. The efficiency of hydrolyze depends on (1) the number of glycoside bonds, which correlates with chain length, (2) the flexibility of chains, and (3) branching of polysaccharide chains. Mannuronic acid is more flexible and more reactive with pepsin than guluronic acid (Chater et al., 2015). Polysaccharides, such as xanthan gum have rigid rod-like chains, which are more resistant to pepsin attack. Branched polysaccharides, such as maltodextrin are more resistant to pepsin attack than unbranched polysaccharide, such as alginate. Pepsin–polysaccharide interactions depend on the charge of polysaccharide under gastric conditions. Interactions between pepsin and polysaccharide molecules are more intensive for molecules with lower value of the isoelectric point. The isoelectric point of (1) xanthan gum is 2.8 (Souza et al., 2013), (2) α-l-gluronic acid is 3.65, (3) β-d-mannuronic acid is 3.38 (Draget et al., 1996), and (4) chitosan is 5.14 (Kalliola et al., 2017). Consequently, short-chain chitosan is more resistant to pepsin attack than alginate because of the higher value of isoelectric point (Boeris et al., 2011). Short chains correspond to lower amount of glycoside bonds per chain, which is desirable.

Efficiency of pepsin attack to proteins depends on (1) their hydrophilic-to-hydrophobic ratio, (2) isoelectric point, (3) the conformation of chains, and (4) interrelation between the mentioned factors under gastric condition. More open conformations enable pepsin attack to aromatic amino acids. β-Lactoglobulin, the major constituent of whey protein, is resistant to pepsin attack in its native form because of its close conformation and low hydrophilic-to-hydrophobic ratio (Lorieau et al., 2018). Contrary, casein and soybean proteins are more reactive with pepsin (Cui et al., 2013; Lorieau et al., 2018).

### Carriers Disintegration Under Gastric Conditions

Lipid carriers represent the best choice in order to keep entrapped active compounds out from the attack of pepsin. Vegetable oils are suitable for the entrapment of EOs, whereas liposomes could be used for immobilization of both hydrophilic and hydrophobic active compounds. Vegetable oils rich by long-chain triglycerides, such as corn oil, nut oil, and canola oil are more suitable because of small digestion rate and resistance to pepsin attack (Majeed et al., 2015).

Polysaccharide hydrogels and polysaccharide–protein composites in the form of microbeads have been widely used for the entrapment of hydrophilic compounds, whereas polysaccharide–protein microbeads have been used for the entrapment of hydrophobic compounds. It is in accordance with the advantage of these carriers, such as (1) easy and cheap preparation procedure and (2) stability under storage conditions in dry state. Ca–alginate have been frequently used for various applications compared with other polysaccharide hydrogels. However, pepsin can easily diffuse through porous structure of amorphous hydrogels and disintegrate them. Hydrodynamic radius of pepsin is 3 nm (Gtari et al., 2017), whereas the average pore size of ionic polysaccharide hydrogel, such as Ca–alginate is in the range from 10 to 20 nm (Funueanu et al., 1999). Diffusion time corresponds to the minute time scale. Grunwald (1989) reported that the internal diffusion of small molecules, such as glucose within 2% Ca–alginate beads was equilibrated during 3 min for a corresponding bead radius of 1.53 mm and during 8 min for corresponding bead radius of 3.20 mm at room temperature.

The process of hydrogel disintegration is much faster than the process of pepsin inactivation by alginate chains. Pepsin inactivation by alginate chains corresponds to the several tens of minutes to hours' time scale (Koutina et al., 2018). The process of hydrogel disintegration corresponds to the diffusion time. In order to improve its stability under gastric condition, Ca–alginate beads have been coated by low-viscosity chitosan (Huguet and Dellacherie, 1996). This is an efficient way to keep hydrophilic active compounds under gastric condition and ensure their transport to low intestine. The blending of Ca–alginate with whey proteins (Zhang S. et al., 2014) improves carriers' resistance to pepsin under gastric condition. Polysaccharide–protein hydrogel carriers consist of (1) polysaccharide phase, which represents the continuum, and (2) dispersed phase, which consists of partially connected protein clusters. Interactions between proteins and polysaccharides at the interface are primarily electrostatic repulsive. The mechanical behavior of these carriers could be understood in the context of two opposite tendencies. Protein clusters are stiffer than surrounding polysaccharide hydrogel. It could be expected that protein addition induces the reinforcement of the carriers. However, both of them are positively charged under gastric condition. Electrostatic repulsive interactions between polysaccharide and protein chains at the interface could induce the weakening of the beads, depending on polysaccharide-to-protein mass ratio.

In summary, most polymeric and oligomeric wrapping structures serving as EOs carriers, such as proteins and carbohydrates, break down by acidic conditions of the gastric phase (Wood, 2005). Digestion rate is directly dependent on the droplet size (Salvia-Trujillo and McClements, 2016), but adversely related to viscosity of dispersion (Ahmad et al., 2018).

Some materials are resistant to gastric digestion (e.g., cellulose and cellulose derivates, resistant starch, some pectins and alginates, etc.) and serve for release of active compounds in the colon, being exposed to subsequent microbial degradation (Belali et al., 2019). Many reports stressed that gut microbial transformation could potentially improve the therapeutic effects of plant bioactive products, including EOs and particular terpenoids (Wang et al., 2019).

In general, the choice of appropriate carrier system and encapsulation technology should be dependent on (1) active ingredient/carrier interaction, (2) bioavailability of the both internal (the active) and the external phase (shell material), (3) release and bioactivity of the active phase at the target site, (4) application needs (e.g., medicine, food, agriculture), (5) safety issues (related to administration route), and (6) sustainability of the entire encapsulation process and economy-based aspects.

## Biomedical Application of Encapsulated EOs and Dependence on Administration Route

Because of potent biological activity and general low genotoxicity and cytotoxicity, the application of EOs in pharmaceutical and cosmetic industry is rapidly increased globally, especially because of expressed multidrug, especially antibiotic resistance, and achievements in drug delivery technologies. Encapsulated EOs or their constituents are used in the form of microparticles and nanoparticles, upon the desired biodegradability of a carrier and projected action site of the active substance.

Topical application of both free and encapsulated EOs is considered as generally safe. Dermal application of microparticles and especially nanoparticles allows the penetration of the active substance into the deeper skin layers providing its sustained and slow release (Bilia et al., 2014). Additional benefits of dermal administration of EOs are that many EO components act as skin penetration enhancers simultaneously exhibiting fast metabolism and excretion rates upon topic application (Herman and Herman, 2014). It was shown that nanoparticles could be used as topical delivery systems for skin cancer treatment (Arpagaus et al., 2018) or as wound healing and skin anti-inflammatory and antiaging agents recently considered as cosmeceuticals (Carvalho et al., 2016). High penetration ability and hydrophobic features of EOs are suitable for their encapsulation in lipid emulsion systems and lipid-based carriers, mainly liposomes that are widely used in dermatology and cosmetics.

Through oral and respiratory route, the delivery systems encounter the mucosal lining of the GI tract and lung, respectively. Therefore, to ensure the target delivery and reaching the action site, the encapsulate should be able to adhere to the mucus, which would enhance the drug absorption and facilitate its transport across the epithelium (Bilia et al., 2014). Because the mucosal surface is negatively charged, the positively charged carriers, such as chitosan, seem to be the most effective (e.g., Kalliola et al., 2017). Furthermore, chitosan is a suitable carrier for mucosal delivery because of low toxicity, good biodegradability, and antibacterial activity (e.g., Kim et al., 2003), in addition to a respiratory application of mannitol, leucine, lactose, and trehalose used because of their high aqueous solubility and low toxicity (Arpagaus et al., 2018).

In regard to achieving the efficient delivery of the active compound into the deep lung regions, the particle size should be designed at 1 to 5 μm, allowing penetration and deposition in the alveolar regions (Arpagaus et al., 2018). The larger particles might be deposited in the throat, whereas the smaller ones are exhaled. It could be expected that application of inhalable EO encapsulated products will increase in the future because of already developed systems for pulmonary drug delivery, including nanoparticles, microparticles (microspheres), solid lipid nanoparticles, and lipid vesicles, such as liposomes (Mehta et al., 2020). It is well-postulated that EOs and their constituents have strong effects on mitigation and healing of many respiratory diseases and disorders (e.g., Horváth and Ács, 2015), so it could be expected that encapsulated inhalable EOs will be favorized over non-encapsulated drugs, because of enhanced bioavailability, better stability, adjustment of dose and optimization of particle size and morphology, and release and lung deposition characteristics.

The biomedical application of encapsulated EOs for oral administration is currently less represented than use in products for human and animal nutrition. However, there are strong benefits of oral intake of encapsulated EOs, referring to great possibility to select the adequate combination of active substance and the prominent biodegradable, edible, and stable carrier for enhanced bioavailability and target site activity. Depending on a goal and the target mode of action, the selection of carrier system would include those sensitive to stomach, the intestine, or colon degradation, as particular EOs exhibit favorable activities at the different final deposition places. The wall materials used for the oral administration of encapsulated EOs should include water-soluble, edible, non-irritant, and biodegradable polymers, such as gelatin, chitosan, maltodextrin, sodium caseinate, pectin, cellulose, gum arabic, alginate, and so on (Arpagaus et al., 2018), and/or their combinations. In our opinion, there is a strong prospect for oral administration of encapsulated EOs, especially in their application against bacterial-caused GI inflammation processes, irritable bowel syndrome, and gastric ulcer conditions. Moreover, the oral intake of encapsulated EOs could be suitable in prophylaxes and for general health improvement. The oral administration of entrapped EOs is the most convenient, the cheapest, and among the safest application routes.

Finally, the intravenous application of encapsulated products is the most questionable administration route, because it is the most delicate and the most risky in terms of safety issues and possible side effects. Despite the fact on low EO toxicity and high biological activity, there are not many reports on their intravenous applications so far. However, the nanoparticles have been already evaluated for the treatment of some solid tumors via intravenous administration (Arpagaus et al., 2018). There is a report on promising systemic delivery of nanoencapsulated linalool in novel cancer therapeutic applications (Han et al., 2016).

Because of enhanced biological ability as a consequence of favorable surface ratio (e.g., Shishir et al., 2018), the nanoparticles are expected to be the main vehicle for the target drug delivery. Nevertheless, the particles at nanoscale exhibit a range of new properties and functionalities of unpredictable effects in the human organism due to potential to penetrate and accumulate much deeper within the human body, thus causing some undesired and untypical effects (De Souza Simões et al., 2017).

### Another Areas for Application of Encapsulated EOs

Apart from entrapment of EOs for target drug delivery, there is wide current application and further possibility for their use in agriculture and the food and textile industry. Upon recent information, the encapsulated EOs and their individual constituents are used in agriculture, mostly as natural pesticides (e.g., Bakry et al., 2016; Kumar et al., 2019) and phytogenic feed additives (e.g., Dajić Stevanović et al., 2018), because of their antibacterial, antifungal, and insecticidal effects. Because of strong antimicrobial activity and expressed fragrance, encapsulated EOs are used in home textiles and personal care products, as well as in production of functional and fragrant textile products (e.g., Bakry et al., 2016).

The application of encapsulated EOs and their constituents is in evident expansion in industry of functional foods and beverages (e.g., Gomez-Mascaraque et al., 2015; Ye et al., 2018; Dima et al., 2020), natural food preservatives and additives (Stratulat et al., 2014; Bakry et al., 2016), and in production of active food packaging as incorporation of these active additives in polymer matrices results in extended food shelf life (Ribeiro-Santos et al., 2017).

There are many-fold benefits of encapsulated EO products application in biomedicine, cosmetics, agriculture, and food and textile industry, referring to enhanced functionality and bioctivity, prolonged shelf life, improved sensory characteristics, increase of systemic activity, and novelty and innovations. However, the main challenges in wider application of such products are the following: safety and regulation issues, achievement of stability and solubility of the active compound, its integration and interfering with other components of a product, possible development of multicapsulation systems for better performances, and the industrial scale-up and high production costs. Finally, there is a need for implementation of green and sustainable technologies in fabrication of encapsulated products.

## Sustainable Production of Carrier Systems for EO Encapsulation

Modern processing biotechnologies should meet the sustainable development requirements and drive industrial competition toward more profitable and innovative way. The original strategies and non-conventional encapsulation methods should address the Green processing concepts (Vinceković et al., 2017), including possibility of reuse and recycling of materials used in encapsulation processes.

Agro-wastes are potential source for recycling polysaccharides, proteins, and lipids and comprise the food wastes and agricultural residues, such as peels and skins, unripe or damaged fruits, seeds, husks, exhausted pulps, and other material. Among the most interesting agro-waste residues as possible polysaccharide sources, the sugarcane and cassava bagasse, the corn stover and straw of corn, oats, wheat, rice, and sorghum may be stressed (Di Donato et al., 2014).

Natural polysaccharides are widely used as a component of carrier matrix applied for various biomedical applications, primarily because of their biocompatibility, ability to form hydrogels, and good mechanical and chemical stability. Pectin, inulin, cellulose, starch, and starch's maltodextrin are frequently used carriers for EOs. Natural polysaccharides for EO carriers are often obtained from wastes of vegetable industrial processing by applying various chemical and enzymatic techniques (Poli et al., 2011). Pectin might be extracted from different waste materials, primarily from the apple pomace, the peel and other by-products of citrus fruit production, and from cherry pomace, pear waste, coffee husk, banana peel, black currant waste, and carrot residues (Di Donato et al., 2014). Inulin is obtained from inulin-rich vegetables and their residues, mainly from leek, onion, garlic, and asparagus, whereas the most common sources are tubers of Jerusalem artichoke and dahlia, in addition to chicory roots (Singh and Singh, 2010). Starch itself is an important carrier for EO encapsulation, which can be obtained from different agro-waste material, such as corn fiber, corn bran, potato peel, and some others (Di Donato et al., 2014). Starch is the only source for maltodextrin production, usually by performing partial acidic or enzymatic hydrolysis. According to Fierascu et al. (2019), high fiber waste of mango rind, broken rice, and pineapple peel and core, as well as red fruits concentrates, are sources of maltodextrin/glucose polymers, other carbohydrates, and simple sugars.

Seaweeds are potential renewable resource of phycocolloids, such as alginate, agar, and carrageenan, where the alginate content may rich 50% of dry weight in some algae, as in *Undaria pinnatifida* (Chee et al., 2011). Alginates are among the most used carriers in encapsulation of bioactives, including EOs, whose chemical structure and related properties differ between genera (McHugh, 2003). Extraction steps focus on converting the alginate to the soluble form of sodium alginate and include stages of pre-extraction with hydrochloric acid, washing, filtration, and neutralization with alkali (Hernandez-Carmona et al., 1999).

The shell and arthropod exoskeloton wastes are rich sources of valuable products, such as calcium carbonate, proteins, carotenoids, and especially chitin, known as the most abundant biopolymer next to the cellulose (Muxika et al., 2017). Extraction of chitin and chitosan requires chemical and fermentation pretreatment processes and application of some intensification techniques, such as ultrasonication and microwave radiation (Suryawanshi et al., 2019).

Polysaccharide matrices are mixed with various proteins in order to improve encapsulation efficiency, chemical stability under gastric condition, and the mechanical stability.

Vegetable proteins, such as those extracted from abundant raw materials (cereals and legumes) or agri-food by-products and waste streams (oilseed meals), have been used as components of carriers. Properties of extracted polymers highly depend on isolation method and conditions. Various techniques for the isolation of proteins, such as micellization technique, alkaline extraction/isoelectric precipitation, ultrasound-assisted extraction, and electroactivation technique and approaches (enzyme-assisted extraction) have been discussed in order to improve protein extraction yield and functionality (Spiegel et al., 2013; Hadnadjev et al., 2017).

The animals' tissues contain proteins that can be used as carriers of bioactive compounds. The most common animal proteins used in encapsulation are collagen, gelatin, and whey proteins (Aspevik et al., 2017; Fathi et al., 2018; Shishir et al., 2018). The meat industry produces significant amounts of collagen waste. Collagen, either collagen fiber or collagen hydrolysate, can be successfully used as a carrier of antioxidants in the food industry or additives in cosmetic products (Mokrejs et al., 2009). Gelatin is a biopolymer produced by partial hydrolysis of collagen derived from animal skin. This type of biomaterial from renewable sources has been frequently used for encapsulation of different types of bioactive compounds (Fathi et al., 2018; Shishir et al., 2018).

**Figure 5:**
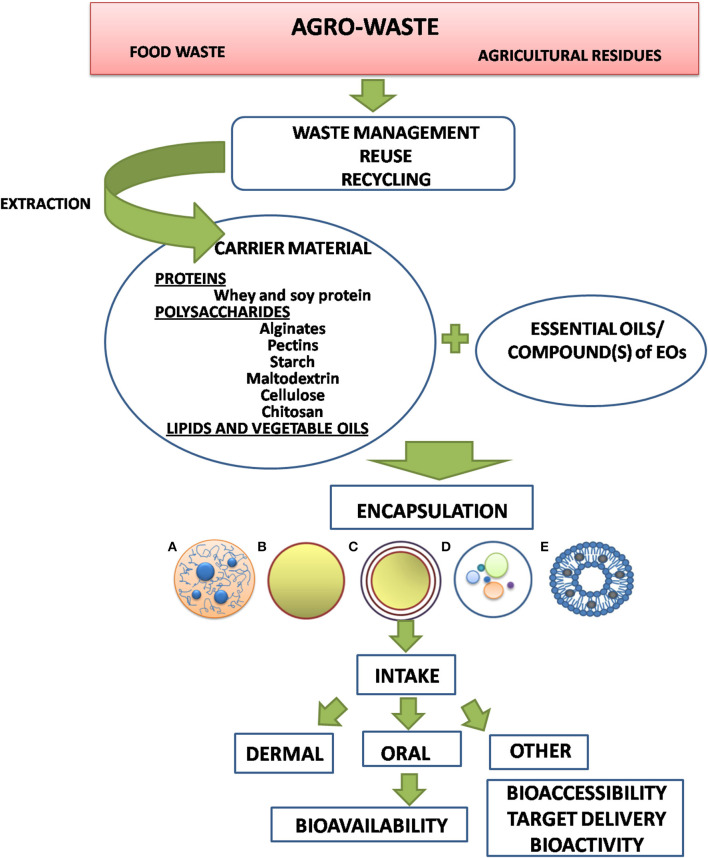
Strategy of choice for the optimal carrier performances of encapsulation of EOs for biomedical application and enhanced bioavailability. **(A)** hydrogel bead, **(B)** monolayer capsule, **(C)** multilayer capsule, **(D)** composite bead, **(E)** liposome.

The dairy industry throughout the world is facing the problem of disposal and utilization of whey. Production of whey proteins by ultrafiltration and the use of whole whey or whey permeate as a fermentation feedstock are possible options to economically recover the valuable nutrients for human food or animal feed. Whey proteins are well-known for their high nutritional and various functional properties in food products. The ability of whey proteins to form gels and microcapsules without the use of severe heat treatment and chemicals makes them an attractive material for controlled delivery applications of bioactive compounds. The bioactive agents (polyphenols, antioxidants, etc.) can be added after the protein denaturation process to minimize the destruction of many of these heat-sensitive components (Wichchukit et al., 2013). The current treatments in whey production rely on application of membrane technologies, such as ultrafiltration, nanofiltration, microfiltration, and inverse osmosis for obtaining the whey powder and whey protein concentrate, demineralized whey powder, and permeate powder (Nicolás et al., 2019). Addition of whey proteins significantly enhances stability of polysaccharide carriers against pepsin attack. Consequently, such type of carrier is suitable for various applications in biomedicine and food technology (Volić et al., 2018; Obradovic et al., 2019). Finally, different reused vegetable oils could be used especially for (nano)emulsion systems, such as sunflower or palm oil for recovery of glycerol, triglycerides, and other lipid compounds used for EO delivery (e.g., Maes et al., 2019), in addition to oils extracted from seeds originating from agro-industrial waste, such as, for example, grape, guava, melon, passion fruit, pumpkin, soursop, and tomato seeds (Silva and da, 2014). In summary, the use of new alternative sources obtained from agro-industrial waste may serve as valuable matrix and carrier material for different encapsulates in food, chemical, and pharmaceutical industries, as was illustrated for encapsulation of EOs in light of the entire EO encapsulation process concept (Figure 5).

## Concluding Remarks

Encapsulation of EOs and their individual compounds is necessary for their target delivery, because of low water solubility and stability, high volatility, and some other unfavorable side effects, such as odor, taste, and sometimes the toxicity. Essential oil activity and toxicity are a result of different interactions of its constituents, such as additivity, synergy, or antagonism. Therefore, it is difficult to predict the overall activity of such complex mixtures as EOs, especially relating their dose and concentration, depending on toxicity effects, frequency of use, and bioavailability. The encapsulation usually prevents the delivery of an EO in the stomach caused by acidic pH and the pepsin attack, but allows the release of the drug in the small intestine. Encapsulation of EOs (1) enables controlled release of bioactive compounds, (2) increases their water solubility and stability, (3) improves the bioavailability and drug efficacy, and (4) reduces eventual toxic effects. Encapsulation is prerequisite of applying EOs in pharmaceutics industry, medicine, and cosmetics, as well as in functional food production. Accordingly, with the fact that EOs and their bioactive compounds exhibit strong antimicrobial and antioxidant activity, there is an increased interest for developing of carriers for delivery of EO-based preservatives in food systems with enhanced chemical, thermal, and oxidative stability. Optimal carriers should be biodegradable, food-grade, and able to protect the EOs from exposure to the surrounding environment prior to release. In case of encapsulation of EOs and their bioavailability, lipid carriers, such as liposomes and solid lipid nanocarriers, in addition to protein–polysaccharide and lipid–protein mixture carriers, should be considered. We would like to emphasize the particular role of protein–polysaccharide hydrogel carriers among others, from the standpoint of (1) the ability of their production from waste materials, (2) simple and cheap encapsulation techniques, (3) satisfied encapsulation efficiency, and (4) good mechanical and chemical stabilities under various *in vivo* process conditions. Finally, it is important to stress a possibility and challenges in use of green biotechnologies, reuse, and waste management practices for obtaining desirable natural carrier macromolecules.

## Author Contributions

ZD coordinated and wrote the part of the manuscript related to biological fate and behavior of EOs and prepared Figures 1 and 5. ES wrote the part of the manuscript related to composition and physicochemical properties of essential oils and prepared Tables 1, 2. KG structured and critically reviewed the entire concept and gave a general insight into all parts of the manuscript. NO wrote the part of the manuscript related to the reuse of agro-waste and prepared Table 3. IP-L wrote the part related to microencapsulation and carriers. All authors contributed to the article and approved the submitted version.

## Conflict of Interest

The authors declare that the research was conducted in the absence of any commercial or financial relationships that could be construed as a potential conflict of interest.

## Footnotes

^1^ISO 9235:2013(en) Aromatic natural raw materials—Vocabulary.
